# The role of zyxin in signal transduction and its relationship with diseases

**DOI:** 10.3389/fmolb.2024.1371549

**Published:** 2024-04-22

**Authors:** Zelan Wu, Daiqin Wu, Qin Zhong, Xue Zou, Zhongjing Liu, Hehua Long, Jing Wei, Xia Li, Fangjie Dai

**Affiliations:** ^1^ Department of Cardiovascular Medicine, The Affiliated Hospital of Guizhou Medical University, Guiyang, China; ^2^ Clinical Research Center, The Affiliated Hospital of Guizhou Medical University, Guiyang, China; ^3^ School of Clinical Laboratory Science, Guizhou Medical University, Guiyang, China; ^4^ Department of Endocrinology, The Affiliated Hospital of Guizhou Medical University, Guiyang, China; ^5^ Guizhou Precision Medicine Institute, Affiliated Hospital of Guizhou Medical University, Guiyang, China

**Keywords:** zyxin, tumorigenesis, cardiovascular diseases, inflammatory responses, signal transduction networks

## Abstract

This review highlighted the pivotal role of zyxin, an essential cell focal adhesions protein, in cellular biology and various diseases. Zyxin can orchestrate the restructuring and dynamic alterations of the cellular cytoskeleton, which is involved in cell proliferation, adhesion, motility, and gene transcription. Aberrant zyxin expression is closely correlated with tumor cell activity and cardiac function in both tumorigenesis and cardiovascular diseases. Moreover, in fibrotic and inflammatory conditions, zyxin can modulate cellular functions and inflammatory responses. Therefore, a comprehensive understanding of zyxin is crucial for deciphering signal transduction networks and disease pathogenesis. Investigating its role in diseases holds promise for novel avenues in early diagnosis and therapeutic strategies. Nevertheless, targeting zyxin as a therapeutic focal point presents challenges in terms of specificity, safety, drug delivery, and resistance. Nonetheless, in-depth studies on zyxin and the application of precision medicine could offer new possibilities for personalized treatment modalities.

## 1 Introduction

Cellular signal transduction, a fundamental regulatory process governing diverse biological functions, operates within a complex network. Zyxin, an integral component of the cellular cytoskeleton, assumes a critical role in this intricate system. It actively participates in interactions with a myriad of proteins, thereby influencing vital cellular processes like adhesion, migration, and growth (Sadler et al., 1992; Nix and Beckerle, 1997; Cerisano et al., 2004; Ermolina et al., 2010; Cattaruzza, 2011; Crone et al., 2011; Rauskolb et al., 2011; Martynova et al., 2013; Wang et al., 2019). Despite these contributions, the exact mechanisms governing zyxin’s participation in signaling pathways remain partially understood.

This review aims to thoroughly investigate zyxin’s role in signal transduction and its connections to significant diseases such as cancer and cardiovascular diseases. By conducting a detailed analysis of zyxin’s structural features, interactions, and functional mechanisms, we aspire to unravel its potential contributions to the pathogenesis of various conditions, including cancer, cardiovascular diseases, fibrotic disorders, and others. Gaining a comprehensive understanding of how zyxin regulates signal transduction and influences disease progression not only enhances our comprehension of pathological mechanisms but also presents opportunities for advancing the development of innovative therapeutic targets.

## 2 Fundamental characteristics of zyxin

### 2.1 Zyxin’s structure and function

Zyxin is an evolutionarily conserved LIM domain protein (Siddiqui et al., 2021), exhibiting significant domain conservation across eukaryotic organisms (Zheng et al., 2016). Expression of zyxin is documented in various organs as per the GeneBank database. It predominantly localizes to focal adhesions (FAs), adherent junctions, and actin stress fibers (Beckerle, 1986; Crawford and Beckerle, 1991; Hirata et al., 2008a). This localization is closely associated with its characteristic LIM domain features. In vertebrates, a variety of proteins with similar structural domains to zyxin are expressed, including TRIP6 (Yi and Beckerle, 1998), LPP (Petit et al., 2000), Ajuba (Goyal et al., 1999), WTIP (Srichai et al., 2004), and LimD1 (Sharp et al., 2004). Zyxin was the first identified mechanically sensitive LIM protein, and it was also the initial protein demonstrated to shuttle between focal adhesions and the cell nucleus (Nix and Beckerle, 1997). Its role in actin dynamics and nucleo-cytoplasmic communication has garnered particular attention (Smith et al., 2014). Zyxin is encoded by the *ZYX* gene in humans (Macalma et al., 1996; Zumbrunn and Trueb, 1996), it consists of 542 amino acids with an unmodified molecular weight of 58,537 (Sadler et al., 1992). Zyxin is a structurally diverse and functionally versatile protein, closely linking its structure to function. It primarily comprises three domains: an N-terminal proline-rich domain (PRD), three C-terminal LIM domains (Sadler et al., 1992), and two nuclear export signals (NES) rich in leucine residues (Smith et al., 2014; Zhao et al., 2022).

#### 2.1.1 Proline-rich domain

It contains multiple repeats rich in proline residues, and this unique structural feature enables zyxin to engage in intricate protein-protein interactions, thereby influencing the reorganization and dynamic changes of the cellular cytoskeleton (Beckerle, 1997). In the field of cell biology, the PRD domain plays a role in various physiological processes, including cell movement, adhesion, and migration. Studies indicate that the PRD domain can interact with a variety of proteins, particularly with SH3 domain-containing proteins (Teyra et al., 2017), actin filament cross-linkers (Reinhard et al., 1999; Li and Trueb, 2001), actin regulatory factors, VASP, and α-actinin (Wang et al., 2019), as well as Ena/VASP (an actin assembly regulator) (Krause et al., 2003). Among these interactions, the selective interaction between the proline-rich sequence in the PRD domain and the SH3 domain is considered the basis for the assembly and arrangement of multiprotein complexes (Teyra et al., 2017). These interactions enable the PRD domain to regulate the assembly and rearrangement of intracellular actin, thereby influencing cell morphology and motility. Researchers have also found that the PRD domain is associated with the cross-linking of actin filaments, contributing to the stability and dynamics of the cellular cytoskeleton. Additionally, through interactions with actin-regulatory factors and Ena/VASP, the PRD domain participates in the regulation of physiological processes such as cell adhesion and migration (Wang et al., 2019).

#### 2.1.2 LIM

The C-terminal LIM domain consists of three zinc finger motifs named after the initial letters of Lin-11, Isl-1, and Mec-3. These motifs accumulate at focal adhesions or force-sensitive sites in the cell, playing a crucial role in their force-sensing functions (Kadrmas and Beckerle, 2004; Steele et al., 2012). Researchers found that only zyxin containing intact LIM domains could accumulate at force-bearing sites, by constructing different zyxin mutants. Further experiments demonstrated that single or truncated LIM domains were unable to accumulate at force-bearing sites. Therefore, all three LIM domains of zyxin are necessary for force-dependent accumulation (Uemura et al., 2011). Simultaneously, the LIM domain is a unique double zinc finger structure with the ability to bind to DNA, proteins, and other nucleic acids, serving as an interface for protein-protein or protein-DNA interactions (Kadrmas and Beckerle, 2004). Furthermore, research has shown that some LIM proteins present in the cell nucleus possess transcriptional functions. In 2003, Wang and Gilmore’s study indicated that the proteins zyxin and paxillin function at focal adhesions in cells, and their LIM domains have functional roles in the cell nucleus (Wang and Gilmore, 2003; Kadrmas and Beckerle, 2004). The LIM structural homology models suggest a high degree of similarity with transcriptional regulatory factors. Further analysis of the surface charge distribution and DNA-binding proteins provides additional support that these LIM domains may be involved in nucleic acid binding (Siddiqui et al., 2021).

#### 2.1.3 Nuclear export signals

Also known as nuclear export signals (NES), located in the central region of zyxin, is a sequence rich in leucine residues. The central region of the zyxin protein contains two NES motifs (Smith et al., 2014). NES can recognize and bind to nuclear transport proteins, precisely regulating the cellular localization of zyxin through interaction with nuclear transport proteins. NES may be involved in signal transduction between focal adhesions and the cell nucleus (Mori et al., 2009; Burridge and Wittchen, 2013), regulating the pathway for nuclear export (Smith et al., 2014). Mutations in NES or treatment of cells with leptomycin B (an inhibitor of Crm1-dependent nuclear export) result in the accumulation of zyxin in the cell nucleus, demonstrating the role of NES in nuclear output in signal transduction (Hervy et al., 2006). In summary, there is a close interrelation between the structure and function of zyxin. Through its various structural domains, zyxin can interact with multiple signaling molecules and proteins, participating in the regulation of various signaling pathways and physiological processes. It plays a crucial role in cell cytoskeleton remodeling, signal activation, and gene transcription.

### 2.2 Role of zyxin in signal transduction

#### 2.2.1 Zyxin and signaling pathways

Signal transduction is intricately associated with focal adhesions. Focal adhesions constitute a high-molecular-weight complex composed of various adhesion proteins, including zyxin, paxillin, vinculin, talin, kindlin, α-actinin, tensin, and focal adhesion kinase (FAK). Their primary function is to establish a connection between cells and the extracellular matrix (ECM), facilitating the transmission of both mechanical and biochemical signals. Focal adhesions serve as centralized hubs for various signaling proteins, guiding and coordinating diverse biological processes, playing a pivotal role (Chen et al., 2003; Ribeiro-Silva et al., 2021). Zyxin is primarily concentrated in actin filaments, stress fiber bundles, and cell-cell or cell-matrix adhesion sites (Crawford and Beckerle, 1991; Sadler et al., 1992), serving as a key component of focal adhesions. Studies have shown that under mechanical stress, zyxin rapidly translocates to actin filaments, while components like vinculin, paxillin, talin, and FAK remain at focal adhesions, suggesting zyxin’s role as a mechanosensitive protein (Yoshigi et al., 2005). Its involvement in actin dynamics and nuclear-cytoplasmic communication has garnered particular attention (Smith et al., 2014). Zyxin plays a crucial connecting role between the extracellular matrix and intracellular signaling pathways. Zyxin is vital for cellular activities, such as cell adhesion, migration, and proliferation (Burridge et al., 1988; Bach, 2000).

#### 2.2.2 Zyxin acts with various proteins

Signal transduction is a precise process that conveys external information into the cell, triggering a series of interconnected molecular events and eliciting cellular responses. These responses can lead to changes in cellular functions, such as promoting or inhibiting cell proliferation, differentiation, migration, and apoptosis. The signal transduction process involves numerous molecules, including receptor proteins, signaling molecules, enzymes, and intracellular and extracellular signaling pathways (physical and chemical). Zyxin not only participates in connecting signaling pathways but can also interact with various proteins, including transcription factors, influencing cellular signal transduction. Additionally, under specific conditions, zyxin can move from cell contact sites to the cell nucleus, directly participating in the regulation of gene expression (Ermolina et al., 2010).

##### 2.2.2.1 Alpha-actinin

Zyxin interacts with various cytoskeletal proteins and regulators, transmitting mechanical signals from the extracellular matrix to intracellular signaling pathways. It regulates cytoskeletal remodeling and stability in response to external forces, influencing cell motility and morphological changes. The actin cytoskeleton of eukaryotic cells plays a central role in various cellular processes, including cell movement, migration, phagocytosis, intracellular transport, and maintenance of cell polarity. Studies have shown that zyxin rapidly relocates from focal adhesions to actin stress fibers in response to mechanical cues (Yoshigi et al., 2005). Zyxin can bind to alpha-actinin in the cell cytoskeleton, promoting cytoskeletal assembly and stability. This interaction is crucial for cell proliferation, movement, migration, and maintenance of cell polarity (Li and Trueb, 2001).

##### 2.2.2.2 Ena/VASP

Additionally, zyxin contains four proline-rich ActA repeats, enabling its interaction with ena/VASP family members (Krause et al., 2003). Ena/VASP is a factor that links actin filament barbed ends and promotes filament elongation (Bear and Gertler, 2009). Proper localization of actin regulatory factors to specific cellular structures and timely regulation are essential for stress fiber response and function. Zyxin binds to ena/VASP, inducing its recruitment to actin filaments, thereby promoting actin assembly and remodeling for mechanically induced stress fiber reinforcement (Yoshigi et al., 2005). Disruption of zyxin-VASP interaction leads to mislocalization of VASP and impaired actin remodeling (Hoffman et al., 2012). Additionally, zyxin interacts with Ena/VASP to regulate E-cadherin-mediated cell-cell adhesion by promoting actin assembly (Nguyen et al., 2010). In summary, zyxin interacts with various cytoskeletal proteins and regulators, regulating cytoskeletal remodeling and stability, and plays a critical role in processes such as cell growth, movement, migration, and morphological changes (Slabodnick et al., 2023).

##### 2.2.2.3 YAP

The ability of cells to respond to mechanical stimuli is crucial for normal physiological function. Cells in the cardiovascular, respiratory, urinary reproductive, and musculoskeletal systems are exposed to mechanical stress under both normal and pathophysiological conditions (Jaalouk and Lammerding, 2009). In the process of mechanical signal transduction, zyxin is considered a significant participant (Beckerle, 1986; Beckerle, 1997; Nix and Beckerle, 1997; Drees et al., 1999; Yoshigi et al., 2005; Hoffman et al., 2006; Hirata et al., 2008a; Hirata et al., 2008b). Zyxin can directly and indirectly control the expression of mechanically sensitive genes (Sporkova et al., 2021), playing a crucial role in mechanical sensing pathways. YAP is a key nuclear transcription factor closely associated with mechanical signals and cell morphology. Studies indicate that under mechanical stimulation, zyxin interacts with YAP, influencing YAP signaling transduction and gene expression (Zhao et al., 2007; Zhang et al., 2023). Zhao et al. found that dynamic stretching along the cell’s long axis activates YAP nuclear translocation, and the knockout of zyxin inhibits YAP nuclear translocation induced by dynamic stretching along the cell’s long axis. Initial cytoskeletal response reveals that stretching along the long axis causes zyxin-mediated actin breakage and repair, regulated by FAK, triggering YAP signaling. However, the specific mechanisms of FAK, zyxin, and YAP interaction remain unknown (Zhao et al., 2007). Zyxin also regulates the expression of pluripotency genes by modulating YAP (Zhang et al., 2023). When cells experience external mechanical stimulation, especially from the extracellular matrix forces, YAP is activated and translocates into the nucleus, regulating the fate of stem cells by controlling the expression of pluripotent genes (Lian et al., 2010; Heng et al., 2020). In the Hippo pathway, phosphorylation of YAP at its S127 site by upstream kinases inhibits YAP activity and retains it in the cytoplasm through interaction with 14-3-3 proteins (Zhao et al., 2007). Experiments with D3 and E14 cell lines by Zhao et al. demonstrate that overexpression of zyxin inhibits YAP nuclear translocation under increased substrate rigidity conditions. In cells overexpressing zyxin, the ratio of phosphorylated YAP to total YAP significantly increases, indicating that Zyxin regulates YAP activity through the mechanical transduction pathway (Zhang et al., 2023). However, the specific mechanisms of zyxin’s interaction with YAP remain to be further studied. In summary, zyxin regulates YAP’s subcellular localization and activity in the mechanical transduction pathway, influencing mechanical transduction and gene expression. This provides a crucial molecular mechanism for determining cell fate, but the exact mechanisms of zyxin’s interaction with YAP require further in-depth investigation (Zhang et al., 2023). Meanwhile, in mammals, YAP/TAZ act as transcriptional co-activators of the Hippo signaling pathway, regulating processes such as cell proliferation, apoptosis, and organ size, playing a critical role in pathway modulation (Oh and Irvine, 2010). Within the Hippo pathway, the counterparts Hippo, Sav, Wts, Mats, and Yki are homologous to mammalian Mst1/2, Sav1, LATS1/2, Mob1, and YAP/TAZ, respectively (Ma et al., 2015). Rauskolb et al. discovered the critical role of the Zyxin protein in regulating both the Fat signaling and the Hippo pathway in their fruit fly model. They found that Zyxin binds with Dachs, subsequently forming a complex with the key protein Wts in the Hippo pathway. This complex promotes Wts degradation, leading to the inhibition of Yki phosphorylation and facilitating Yki accumulation in the cell nucleus. This regulation ultimately affects cell proliferation and organ size (Rauskolb et al., 2011). This research confirms the association between Zyxin and the Hippo pathway, providing significant insights into understanding the mechanisms governing cell proliferation and organ development. Under hypoxic conditions and TGF-β stimulation, Zyxin forms a ternary complex with Lats2 and Siah2. This interaction promotes Lats2 ubiquitination and degradation, as well as YAP dephosphorylation, subsequently activating YAP and facilitating its nuclear translocation, thereby regulating gene expression. Experimental evidence shows that Zyxin depletion leads to decreased cell proliferation rates and reduces tumor volume and weight. These findings underscore the regulatory role of Zyxin in the Hippo and TGF-β signaling pathways (Ma et al., 2016).

On the other hand, under mechanical force stimulation, zyxin may potentially enter the cell nucleus along with other proteins, and subsequently exit the nucleus through an internal NES rich in leucine residues. Due to zyxin’s ability to shuttle between the cytoplasm and the cell nucleus, it may potentially mediate cellular functions in a force-dependent manner. Its capability to sense mechanical force might be a crucial component in the regulation of gene expression (Nix and Beckerle, 1997; Uemura et al., 2011). For instance, in vascular cells, supraphysiological levels of stretch can induce the translocation of zyxin to the cell nucleus, promoting gene expression under stretching stimuli (Suresh Babu et al., 2012). Stretching stimuli induce the release of Endothelin-1 (ET-1) via Transient Receptor Potential Channel 3 (TRPC3) mediated pathways, which, upon autocrine activation, triggers the release of Atrial Natriuretic Peptide (ANP), leading to the phosphorylation of zyxin at serine 142 by protein kinase G and subsequent translocation of zyxin to the cell nucleus (Suresh Babu et al., 2012).In this context, zyxin is essential for stretch-induced gene expression, serving as a mediator for transmitting mechanical stimuli signals within the cell and participating in the regulation of gene expression (Suresh Babu et al., 2012).

##### 2.2.2.4 Homeodomain-interacting protein kinase 2

The study also reveal ed the association of zyxin with the homeodomain-interacting protein kinase 2 (HIPK2). HIPK2 is a critical regulatory factor in cell fate and plays a crucial role in the cellular response to genomic damage. For instance, in non-stressed cells, the levels of HIPK2 are maintained at very low levels. However, under stressful conditions such as exposure to ultraviolet radiation, irradiation, or chemotherapy causing severe genomic damage, HIPK2 is activated. Subsequently, p53 undergoes phosphorylation, leading to apoptosis (Bitomsky and Hofmann, 2009). Additionally, research has demonstrated that the presence of zyxin can influence the stability of the HIPK2 protein, thereby affecting the phosphorylation of p53 at serine 46, a crucial apoptosis signal. Specifically, zyxin can interact with the Siah-1, an enzyme responsible for mediating the degradation of HIPK2 (Kim et al., 2009), regulating the activity of Siah-1 and consequently impacting the degradation of HIPK2. The stability of HIPK2 directly influences the phosphorylation of p53 and the activation of apoptosis pathways associated with it. Therefore, the presence of zyxin can modulate the activity of the HIPK2-p53 signaling pathway, playing a significant role in the determination of cell fate induced by DNA damage (Crone et al., 2011). This discovery reveals a novel role for zyxin in the regulation of cellular life and death, providing a fresh perspective for a deeper understanding of cellular stress responses. On the other hand, HIPK2 can directly bind to and phosphorylate β-catenin, leading to its degradation, thereby regulating the Wnt/β-catenin signaling pathway (Kim et al., 2010). This implies that Zyxin may further participate in the regulation of the Wnt/β-catenin signaling pathway by modulating the stability of HIPK2.

##### 2.2.2.5 AKt

Multiple studies have demonstrated that, under the influence of Protein Kinase B (Akt), phosphorylation occurs at the 142nd amino acid of the zyxin protein, facilitating its interaction with the 14-3-3γ protein. Consequently, zyxin protein translocates from the cytoplasm to the nucleus. This suggests a crucial role for zyxin in cellular signal transduction and regulation, participating in cellular responses and control by translocating between the nucleus and cytoplasm (Nix and Beckerle, 1997; Chan et al., 2007). In the study conducted by Kato et al., 2005, it was found that Atrial Natriuretic Peptide (ANP) promotes the survival of myocardial cells through the cGMP-dependent accumulation of nuclear zyxin and Akt proteins. The nuclear translocation of zyxin also triggers the accumulation of activated Akt kinase in the cell nucleus. Zyxin and activated Akt participate in a cGMP-dependent signaling cascade. Overall, the accumulation of zyxin and activated Akt in the cell nucleus may represent a fundamental mechanism promoting nuclear signal transduction and enhancing cell survival (Kadrmas and Beckerle, 2004). Additionally, another study suggests that zyxin interacts with acinus-S, a nuclear speckle protein that induces apoptosis and chromatin condensation upon caspase cleavage. Zyxin can inhibit the apoptotic effect of acinus-S, and this inhibition is regulated by Akt (Chan et al., 2007).

##### 2.2.2.6 SIRT1

Zyxin may serve as a novel interacting partner with SIRT1. Researchers employed yeast two-hybrid technology to screen from a human embryonic brain cDNA library and identified a potential binding between SIRT1 and zyxin. Transcripts of SIRT1 and zyxin are abundantly expressed in the developing mouse brain. In COS-7 cells, zyxin accumulates in the nucleus after treatment with leptomycin B, co-localizing with SIRT1. Furthermore, SIRT1 is capable of deacetylating zyxin, indicating that SIRT1 may interact with nuclear-accumulated zyxin through deacetylation, influencing its function. In conclusion, research findings suggest that zyxin may serve as a novel binding partner for SIRT1. As an adapter protein, zyxin plays a role in regulating signal transduction and cytoskeletal dynamics in cell adhesion plaques. SIRT1, on the other hand, appears to modulate zyxin’s function through deacetylation, thereby influencing the signaling cascade from the extracellular matrix to the cell nucleus (Fujita et al., 2009).

##### 2.2.2.7 PAR-1

Experiments suggest that zyxin plays a role in signaling by binding to the C-terminal domain of PAR-1, participating in thrombin-induced actin cytoskeleton remodeling, and SRE-dependent gene transcription. Disruption of the PAR-1-zyxin interaction inhibits stress fiber formation and activation of the Serum Response Element (SRE). Depletion of zyxin also affects the recovery capacity of the endothelial barrier. Additionally, zyxin may be involved in recruiting VASP to regulate thrombin-induced cytoskeletal remodeling (Han et al., 2009). This study provides evidence for a novel signaling pathway mediated by zyxin in the thrombin signaling pathway through its interaction with PAR-1.

**FIGURE 1:**
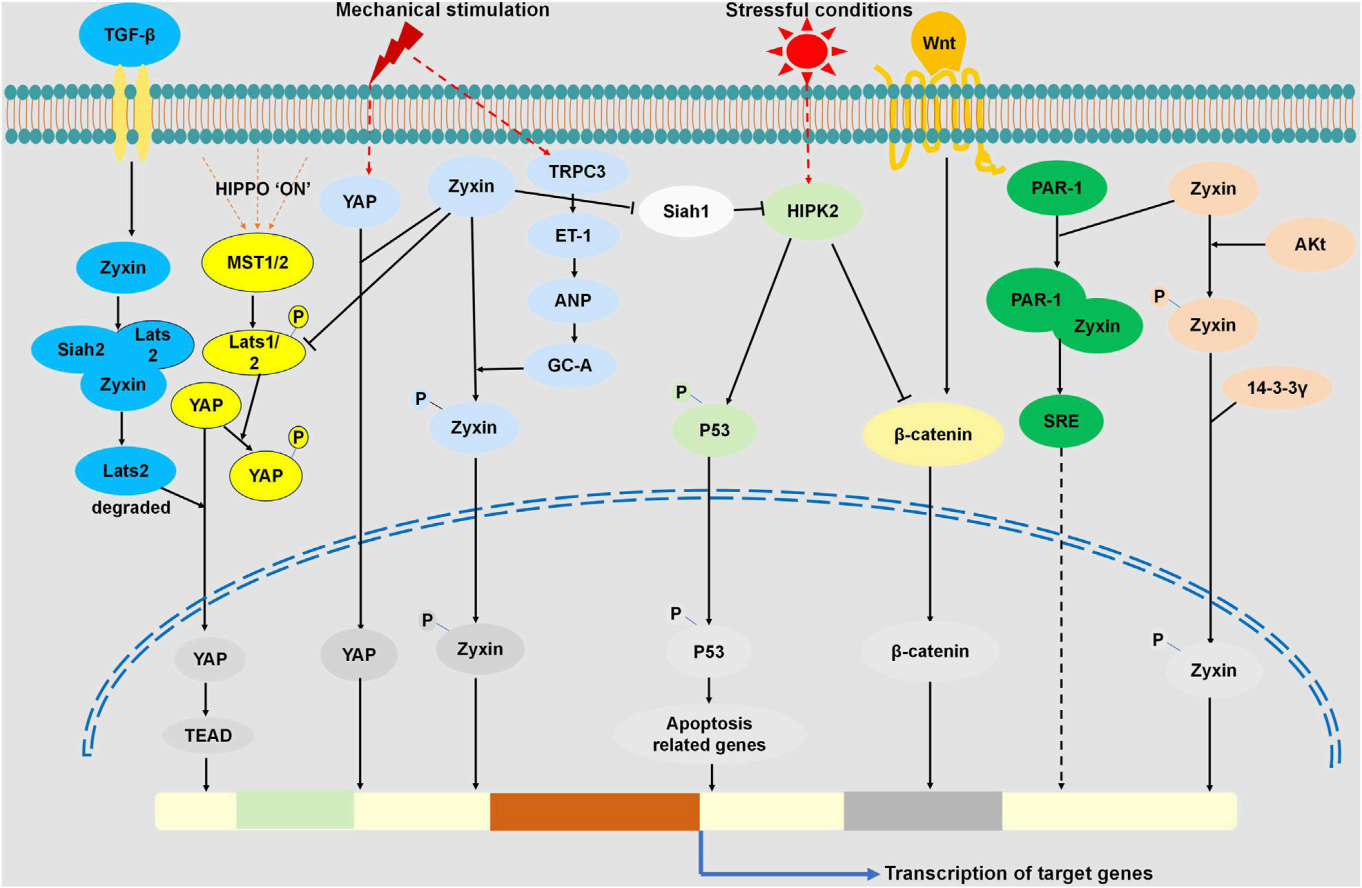
Zyxin Regulated Signaling Pathways through Interactions with Multiple Proteins. Cellular response to mechanical stimuli activates YAP, promoting its nuclear translocation for gene expression. Zyxin interacts with YAP, influencing mechanosensitive pathways. In the Hippo pathway, Zyxin binds Dachs, forming a complex with LATS1/2, promoting LATS1/2 degradation, inhibiting LATS1/2 phosphorylation, consequently suppressing YAP phosphorylation. This facilitates YAP nuclear translocation, regulating the Hippo pathway. Under TGF-β stimulation, Zyxin forms a ternary complex with Lats2 and Siah2, promoting Lats2 ubiquitination and degradation. This enhances YAP dephosphorylation and nuclear translocation, regulating the TGF-β pathway. Stretch stimulation activates TRPC3, leading to ET-1 release, triggering ANP release. Consequently, GC-A is activated, initiating the protein kinase G-mediated zyxin phosphorylation and nuclear translocation. Under stress, HIPK2 activation induces p53 phosphorylation and apoptosis. Zyxin interacts with Siah-1, suppressing its activity, preventing HIPK2 degradation, and enhancing p53 phosphorylation, activating the HIPK2-p53 apoptotic pathway. HIPK2 binds to and phosphorylates β-catenin, promoting its degradation, inhibiting nuclear translocation, regulating Wnt/β-catenin signaling.Upon Akt activation, zyxin’s 142nd amino acid undergoes phosphorylation, binding with 14-3-3γ, causing nuclear translocation. Zyxin binds PAR-1, promoting SRE activation, contributing to signal transduction in SRE-dependent gene transcription.

In conclusion, Zyxin regulates multiple signaling pathways through interactions with various proteins (Figure 1), providing novel insights into cellular responses to mechanical stimuli and stress. Its involvement in key pathways such as Hippo, TGF-β, and Wnt/β-catenin signaling, influencing nuclear translocation and gene expression. Moreover, Zyxin modulates TRPC3, HIPK2-p53, and Akt pathways, revealing its multifaceted role in cellular signaling. These novel aspects underscore the significance of zyxin as a central regulator in cellular mechanotransduction and stress response pathways, suggesting its potential as a therapeutic target for various diseases.

In addition, zyxin can interact with Protein Kinase C (PKC) to regulate cell growth and behavior. It can interact with Bcl-2, affecting cell survival and anti-apoptotic ability (Bernusso, 2013). It can also interact with Src kinase, enhancing or inhibiting cell proliferation (Sporkova et al., 2021). Zyxin can also interact with various transcription factors, such as 6E6 (Degenhardt and Silverstein, 2001), HNF-1β (Hiesberger et al., 2004), CARP-1 (Hervy et al., 2010), Xanf1 (Martynova et al., 2008), ZNF384 (Janssen and Marynen, 2006), CBP (Youn et al., 2013), influencing various biological functions of the cell (Table 1).

**TABLE 1:** Other proteins interacting with zyxin.

Proteins	Effect	References
alpha-actinin	Influencing cell motility and morphology	Slabodnick et al. (2023)
Ena/VASP	Promoting actin assembly and remodeling	Yoshigi et al. (2005)
LASP-1	Influencing cell spreading and proliferation	Ma et al. (2016)
PKC	Regulating cell growth and behavior	Nishizuka (1995)
acinus-S	inhibiting the apoptotic effect of acinus-S	Chan et al. (2007)
STRT1	Regulating extracellular matrix to the cell nucleus signaling	Fujita et al. (2009)
Bcl-2	Influencing cell survival and anti-apoptotic capability	Bernusso (2013)
Src	Regulating cell proliferation	Sporkova et al. (2021)
6E6	Promoting zyxin nuclear translocation for transcriptional activation	Degenhardt and Silverstein (2001)
HNF-1β	Regulating gene expression in organs such as liver, kidney, and intestine	Hiesberger et al. (2004)
CARP-1	Regulating UV-induced cell apoptosis	Hervy et al. (2010)
Xanf1	Regulating brain development	Martynova et al. (2008)
ZNF384	Regulating bone metabolism	Janssen and Marynen (2006)
CBP	Regulating RAR activity, influencing the efficacy of retinoic acid therapy	Youn et al. (2013)

In summary, zyxin not only acts as a mediator between the extracellular matrix and intracellular signaling pathways but also integrates mechanical and chemical signals by interacting with various proteins and transcription factors. Its roles in crucial cellular processes such as adhesion, migration, and signal transduction highlight its significance. Despite remaining gaps in our understanding of the specific mechanisms, further research holds the promise of unraveling the intricate functions and regulatory pathways of zyxin in cell signaling. This continued exploration may unveil potential clinical applications of zyxin in the future.

### 2.3 Zyxin and embryonic development

The exact role of zyxin in embryonic development remains incompletely understood, but several studies have provided clues. Knocking down zyxin mRNA levels in avian embryos revealed its involvement in regulating cell migration and differentiation during the formation of atrioventricular valves (Li et al., 2013), suggesting a potential key role in embryonic heart development. Additionally, knockout of zyxin in the nematode *C. elegans*, lacking a zyxin homolog, demonstrated its role in regulating mechanosensation at neuronal synapses (Luo et al., 2014). In another invertebrate model, *Drosophila melanogaster*, widespread downregulation of zyxin resulted in incomplete air-filling of adult fly tracheae, further confirming its importance in normal development (Renfranz et al., 2010). However, no abnormal phenotypes were observed upon zyxin gene knockout in mice (Hoffman et al., 2003), which may be attributed to genetic compensatory mechanisms (Rossi et al., 2015). Although zyxin knockout mice can develop and grow normally (Hoffman et al., 2003), under experimental hypertension conditions, they exhibit more pronounced abnormalities in cardiac contractile function, increased apoptosis, and exacerbated fibrosis compared to wild-type mice (Nahar, 2017; Al-Hasani et al., 2022). This indicates the crucial role of zyxin in responding to physiological/pathological stressors, while genetic compensatory mechanisms may mask some effects of zyxin deficiency under normal conditions, necessitating further research for clarification.

While we have noted that disrupting Zyxin expression could affect specific facets of embryogenesis in animal models, the exact contribution of Zyxin to human embryonic development remains inadequately comprehended. Consequently, additional experimental investigations are imperative to illuminate its function and mechanisms in human embryonic development. At present, we aim to delve further into examining the correlation between Zyxin and diverse pathological conditions.

## 3 Association of zyxin and disease

As previously discussed, zyxin plays a pivotal role in various cellular functions, encompassing the assembly of focal adhesions, organization of the actin cytoskeleton, regulation of gene expression, as well as pivotal processes like cell growth, proliferation, and migration. The aberrant expression or dysfunction of zyxin is implicated in the onset and progression of several diseases.

### 3.1 Zyxin and tumor

Zyxin plays a crucial role in the invasion and metastasis processes of tumors. Its association with cell migration, invasion, and potential therapeutic targets has garnered significant attention in cancer research. Recent studies have confirmed the substantial involvement of zyxin in the development of various types of cancers (Van der Gaag et al., 2002; Grunewald et al., 2007; Mise et al., 2012; Yamamura et al., 2013; Ma et al., 2016; Zhao et al., 2016). Zyxin may play a dual role as an oncogene or a tumor suppressor in cancer diseases. Researchers have observed that in certain tissue sites, zyxin could act as an oncogene, promoting the development and progression of cancer. However, in other organs, it might function as a tumor suppressor, inhibiting the advancement of cancer. This dual role could be influenced by factors such as the type of cancer, specific organs involved, experimental conditions, and the presence or absence of interacting proteins (Kotb et al., 2018).

In terms of promoting tumors, studies indicate that in melanoma cells, the expression of zyxin, focal adhesion kinase (FAK), and paxillin increases, directly correlating with cell spreading and proliferation but showing a negative correlation with differentiation. Drug treatment experiments demonstrate that modifying zyxin expression can impact cell behavior (Van der Gaag et al., 2002). In 2008, Wagner et al. confirmed the association between zyxin and melanoma through their study of the Wilms tumor protein (WT1) in relation to melanoma. They found that in malignant melanoma, over 80% of cells express WT1, while it is not expressed in normal skin or benign nevi. Silencing WT1 resulted in decreased zyxin expression, leading to reduced proliferation of melanoma cells (Wagner et al., 2008). These findings suggest that developing new strategies for melanoma treatment may involve regulating zyxin. Additionally, studies have shown that zyxin is overexpressed in some cases of hepatocellular carcinoma (HCC), especially in cases of multiple tumors, where the proportion of zyxin overexpression can be as high as 60-fold (Sy et al., 2006). Zyxin promotes the malignant progression of HCC by activating the AKT/mTOR signaling pathway (Cai et al., 2023), making it a potential therapeutic target for HCC. Overexpression of zyxin has also been observed in malignant renal tumors (Gianazza et al., 2012). In breast cancer, approximately 70% of breast cancer tissues express zyxin, whereas normal breast tissues have an expression rate of only about 5%. Zyxin directly influences cell spreading and proliferation by binding to the LASP-1 protein, thereby promoting the development and progression of breast cancer (Ma et al., 2016).

In terms of tumor suppression, zyxin is known to bind to Cell Cycle and Apoptosis Regulatory Protein 1 (CARP-1). The LIM domain of zyxin, which binds to CARP-1, is a crucial region for the pro-apoptotic function of zyxin (Hervy et al., 2010). Research indicates that zyxin interacts with Myopodin, exerting inhibitory effects on prostate cancer/testicular cancer (Yu and Luo, 2006; Korkola et al., 2009). Zyxin also exhibits inhibitory effects on bladder cancer by influencing the β-catenin signaling pathway. β-catenin is a cytoplasmic protein that interacts with proteins such as zyxin, E-cadherin, and moesin, participating in the assembly of adhesive connections between cells. Under normal physiological conditions, the interaction of β-catenin with proteins like zyxin, E-cadherin, and moesin maintains a balance in cell adhesion connections both inside and outside the cell. However, in diseases like bladder cancer, this balance may be disrupted, leading to increased invasiveness of cells (Shiina et al., 2001). The exact mechanism of zyxin in bladder cancer, though, requires further research for a detailed understanding. In Ewing sarcoma, a downregulation of zyxin expression levels has been observed. This phenomenon may be attributed to the repositioning of zyxin within the cytoplasm rather than at cell adhesion sites in this type of sarcoma. Further research indicates that this redistribution might result from abnormal translocation of the zyxin gene, leading to the reorganization of the actin cytoskeleton. This process further diminishes the vitality and growth capacity of tumor cells (Amsellem et al., 2005). In this sarcoma, zyxin seems to act as a tumor suppressor. Its abnormal localization and downregulated expression levels may be contributing factors to impaired functionality of tumor cells, especially in association with the abnormal reorganization of the actin cytoskeleton. However, more research is needed to gain a deeper understanding of the specific mechanisms through which zyxin operates in Ewing sarcoma and its molecular events related to the occurrence and development of other tumors.

To summarize, multiple studies suggest that zyxin plays a crucial role in the development of cancer. However, it should be noted that the above content is based on existing research results and theoretical speculation. Further experiments and clinical studies are needed to validate and explore the exact role and clinical significance of zyxin in cancer. Understanding the function and regulatory mechanisms of zyxin in-depth could contribute to the development of novel therapeutic strategies for cancer treatment and offer personalized treatment approaches.

### 3.2 Zyxin and cardiovascular disease

Zyxin’s relationship with cardiovascular diseases requires further comprehensive research for a thorough understanding. Some studies suggest that zyxin may play a role in the occurrence and development of cardiovascular diseases. A study utilizing zyxin gene knockout mouse models investigated the impact of zyxin on heart function under experimental hypertension conditions. The results indicated that, under high blood pressure conditions, mice with zyxin gene knockout exhibited abnormalities such as impaired cardiac contraction, increased apoptosis, and intensified fibrosis. This research suggests that zyxin plays a crucial role in maintaining heart function under hypertensive conditions (Al-Hasani et al., 2022). These findings reveal the involvement of zyxin in the pathogenesis of cardiac diseases, providing new insights into the mechanisms underlying the development of heart diseases.

Moreover, studies in zyxin knockout mouse models have indicated that under hypertensive conditions, vascular smooth muscle cells (VSMCs) maintain a contractile phenotype through the collaborative action of zyxin and Lipoma Preferred Partner (LPP), thereby preventing the development of vascular remodeling. Zyxin also has a positive impact on cardiomyocytes, and its deficiency may lead to cardiomyocyte apoptosis and excessive fibrosis, thereby affecting heart function (Nahar, 2017). Studies have further highlighted the crucial role of zyxin in regulating excessive collagen deposition in fibroblasts (Nahar, 2017). In general, these studies provide in-depth insights into the crucial roles of zyxin and LPP in regulating hypertension-induced cardiovascular remodeling, offering new clues for a better understanding and intervention in cardiovascular diseases.

Furthermore, zyxin also plays a significant role in atherosclerosis. On one hand, when the mechanical environment of vascular cells changes, zyxin, acting as a specific mechanical sensor protein, is activated, triggering a series of signaling events. This activation process involves multiple steps: firstly, through TRPC3-mediated release of ET-1, ET-1 activates the release of atrial natriuretic peptide (ANP) through autocrine B-type receptor stimulation, and then ANP, by activating the receptor GC-A/cGMP/protein kinase G pathway, phosphorylates zyxin at serine-142. This phosphorylation enables zyxin to translocate to the cell nucleus, binding to a novel stretch-sensitive promoter sequence PyPu-box, influencing the expression of approximately 70% of stretch-sensitive genes in endothelial cells and smooth muscle cells. The nuclear translocation and gene regulation processes of zyxin may be involved in the development and progression of atherosclerosis (Cattaruzza, 2011). On the other hand, zyxin, by regulating the gene expression of endothelial cells, promotes the inflammatory response of endothelial cells, leading to endothelial dysfunction and the occurrence of atherosclerosis (Wójtowicz et al., 2010).

Additionally, research has delved into the role of zyxin in myocardial hypertrophy. The cytoplasmic domain of β1 integrin and vinculin are critical structures necessary for maintaining proper muscle architecture, heart development, and function, Even minor changes can lead to defects in the hypertrophic response to mechanical load (Brancaccio et al., 2006). Zyxin, FAK, Melusin, and signaling proteins such as ENA/VASP are essential for the correct hypertrophic response when the heart is subjected to hemodynamic overload (Brancaccio et al., 2006). Specifically, β1 integrin serves as a connector between the extracellular matrix and the intracellular actin cytoskeleton, regulating cell adhesion and signaling. Vinculin, as a structural protein, maintains cell integrity and forms a connection with β1 integrin, anchoring actin filaments to the cell membrane. Zyxin, functioning as a signaling protein, interacts with β1 integrin and vinculin, participating in the modulation of cell function and morphology (Brancaccio et al., 2006). Therefore, they represent potential genetic modifiers, and mutations in these genes may increase susceptibility to myocardial diseases (Brancaccio et al., 2006). Furthermore, in the study by Joshua et al., higher levels of Zyxin were found in the failing hearts of Dilated Cardiomyopathy (DCM) (DeAguero et al., 2017). However, its exact mechanism of action remains unclear.

Although there have been some studies regarding the relationship between zyxin and cardiovascular diseases, these studies are primarily limited to animal models. More research and experimental evidence are needed to further elucidate the exact role and mechanisms of zyxin in cardiovascular diseases.

### 3.3 Zyxin and fibrotic disease

Fibrotic diseases refer to conditions characterized by excessive activation of fibroblasts and collagen deposition in tissues or organs, leading to abnormal tissue structure proliferation and fibrosis. Current research is still exploring the precise mechanisms and therapeutic potential of zyxin in fibrotic diseases. The research indicates that zyxin plays a key role in fibroblast stiffness sensing and durotaxis, potentially influencing the function of fibroblasts (Yip et al., 2021). By reanalyzing high-throughput RNA sequencing data, researchers have identified that the key protein zyxin plays a crucial role in skin fibrosis, participating in the regulation of extracellular matrix deposition. Further validation revealed upregulation of zyxin expression in skin tissues of various fibrotic diseases (Huang et al., 2023). Confirmation through zyxin knockout mice, nude mouse models, and human systemic sclerosis (SSc) scar tissue cultures demonstrated that zyxin inhibition significantly alleviates skin fibrosis (Huang et al., 2023). Dual immunofluorescence staining showed high expression of zyxin in fibroblasts (Huang et al., 2023). Additional analysis indicated that zyxin overexpression increased the expression of fibrotic genes and collagen production in fibroblasts, while disruption of zyxin expression reduced these processes in SSc fibroblasts. Transcriptome and cell culture analyses revealed that zyxin inhibition effectively mitigates skin fibrosis by modulating the FAK/PI3K/AKT and TGF-β signaling pathways through integrins (Huang et al., 2023). These studies indicate that zyxin might play a significant role in regulating fibrotic diseases.

While more research is needed to determine the potential of zyxin in the treatment of fibrotic diseases, preliminary experimental results suggest that targeting zyxin could have therapeutic potential for fibrosis. For instance, interference with Zyxin significantly alleviates fibrosis in scarred skin tissue explants, reducing collagen content. (Huang et al., 2023). Zyxin could emerge as a potential novel target for treating skin fibrosis.

### 3.4 Zyxin and other diseases

In addition to the aforementioned diseases, there has been some progress in the research on zyxin in other disease areas.

#### 3.4.1 Inflammatory diseases

Inflammatory diseases such as psoriasis (Plavina et al., 2008), inflammatory bowel disease (Dai et al., 2022) are associated with the expression and regulation of zyxin. Psoriasis, a chronic inflammatory skin disease, exhibits increased zyxin levels in patients’ plasma (Plavina et al., 2008). Although its precise mechanism remains unclear. Additionally, in ulcerative colitis, zyxin (ZYX), RhoB, and cathepsin D (CTSD) have been identified as core genes through single-cell RNA sequencing (scRNA-seq) analysis. Further functional enrichment analysis indicates a close association of these differentially expressed genes with immune and inflammatory responses, potentially serving as diagnostic markers and molecular targets for UC therapeutic intervention (Dai et al., 2022). Integrating these findings, zyxin appears to be involved in the occurrence and progression of inflammatory diseases. However, further research and clinical trials are needed to validate its role and potential therapeutic implications.

#### 3.4.2 Neurological disorders

Research has shown that zyxin is involved in neuronal information transmission, morphological regulation, and the maintenance of synaptic function (Fujita et al., 2009; Luo et al., 2014). In the case of neurodegenerative diseases such as Alzheimer’s disease (AD), the abnormal expression and dysfunction of zyxin may be associated with the onset and progression of the disease. Using HEK-293 and SH-SY5Y cell models, it was found that β-amyloid 1–40 and 1–42 led to zyxin dysregulation, disrupting the transcriptional inhibitory activity of HIPK2 on its target genes, resulting in changes in the conformational state of p53. This reveals zyxin as a novel target for Aβ activity. This discovery is expected to deepen the understanding of the pathogenesis of AD and provide a new perspective for its treatment (Lanni et al., 2013). In addition, recent research has used a genetic mutation burden analysis approach to identify the ZYX gene, which encodes the zyxin protein, as a new candidate gene in the pathogenesis of Moyamoya disease. This suggests that zyxin may be associated with the pathogenic mechanism of Moyamoya disease (Barak et al., 2023). However, the specific mechanisms of action and effects still require further research for clarification.

#### 3.4.3 Hematological diseases

Research using zyxin-deficient mouse models has revealed severe macrothrombocytopenia resulting from zyxin deficiency. Proteomic analysis showed a significant reduction in Glycoprotein Ib-IX (GPIb-IX) protein due to zyxin deficiency. Zyxin colocalizes with VASP, and zyxin loss leads to disruption of VASP and actin cytoskeleton. Reconstitution of zyxin with the VASP binding site restores GPIb-IX surface expression and platelet generation. Thus, zyxin regulates platelet biogenesis and GPIb-IX surface expression through VASP-mediated cytoskeleton reorganization, uncovering potential mechanisms of macrothrombocytopenia.

It should be noted that, despite some studies suggesting the potential role of zyxin in the mentioned disease areas (Table 2), further experiments and clinical research are needed to validate and confirm these results. For each specific disease, the function and regulatory mechanisms of zyxin may vary. Therefore, a thorough understanding and investigation of the role of zyxin in different diseases will help reveal its potential therapeutic value and provide new insights and strategies for the diagnosis and treatment of diseases.

**TABLE 2:** Diseases associated with zyxin.

Disease		Effects of zyxin in disease^a^	References
Tumor	Malignant melanoma	+	Van der Gaag et al. (2002), Wagner et al. (2008)
	Hepatocellular carcinoma	+	Cai et al. (2023)
	Malignant renal tumor	+	Gianazza et al. (2012)
	Breast cancer	+	Ma et al. (2016)
	non-Hodgkin’s lymphoma	+	Miao et al. (2016)
	Glioma	+	Lin et al. (2013)
	Prostate cancer	-	Yu and Luo (2006)
	Bladder cancer	-	Shiina et al. (2001)
	Ewing sarcoma	-	Amsellem et al. (2005)
cardiovascular disease	Hypertensive heart disease	-	Al-Hasani et al. (2022)
	Atherosclerosis	+	Cattaruzza et al. (2004)
	Dilated cardiomyopathy	+/−	DeAguero et al. (2017)
fibrotic disease	Skin fibrosis	+	Huang et al. (2023)
Inflammatory diseases	Psoriasis	+/−	Plavina et al. (2008)
	Inflammatory bowel disease	+	Dai et al. (2022)
Neurological disease	Alzheimer’s disease	+	Lanni et al. (2013)
	Moyamoya	+/−	Barak et al. (2023)

^a^ Zyxin promotes disease progression indicated by “+”, inhibits disease progression indicated by “-”, and has an effect that remains unclear indicated by “+/−”.

## 4 Potential and challenge of zyxin as a therapeutic target

Zyxin plays a pivotal role in regulating the structure and function of cells, involving key processes such as cell adhesion, migration, and proliferation. Therefore, by modulating the activity and function of zyxin, it is possible to influence the occurrence and development of various diseases. Zyxin participates in the regulation of multiple signaling pathways, including the Wnt/β-catenin (Kim et al., 2010), TGF-β (Mise et al., 2012), Hippo (Rauskolb et al., 2011), and other common cellular signaling pathways. Targeting the regulation of zyxin may potentially intervene in the abnormal activity of these signaling pathways, thereby influencing the occurrence and progression of related diseases. This indicates that zyxin, as a therapeutic target, may have potential and broad application value. However, it also faces some challenges.

### 4.1 Potential of zyxin as a therapeutic target

#### 4.1.1 Cancer regulation

Zyxin exhibits potential as a therapeutic target in the field of cancer. As mentioned earlier, Zyxin is involved in regulating cell adhesion, migration, and invasion (Burridge et al., 1988; Ermolina et al., 2010). By influencing these critical processes, it may have an impact on the infiltration and metastasis of cancer cells, making it a potential target for cancer treatment.

#### 4.1.2 Cardiovascular disease regulation

Zyxin plays a regulatory role in cardiomyocytes within the cardiovascular system. Lack of Zyxin may lead to cardiomyocyte apoptosis and excessive fibrosis, promoting myocardial remodeling and affecting heart function (Nahar, 2017; Al-Hasani et al., 2022). This suggests that Zyxin could have potential implications in the treatment of cardiovascular diseases, emerging as a novel area for regulating cardiomyocyte function.

#### 4.1.3 Fibrotic disease regulation

In fibrotic diseases, Zyxin plays a crucial role in the activation of fibroblasts and collagen deposition. Inhibiting Zyxin’s function in fibroblasts may reduce collagen deposition and mitigate the occurrence of fibrosis (Huang et al., 2023). Zyxin holds the potential to become a promising new target for treating skin fibrosis.

#### 4.1.4 Inflammatory diseases regulation

Zyxin’s association with inflammatory diseases has also been under investigation. For example, studies have shown that zyxin (ZYX) is one of the core genes in ulcerative colitis, regulating its gene expression, and is expected to become a therapeutic target for ulcerative colitis (Dai et al., 2022).

#### 4.1.5 Neurological disorders regulation

Zyxin is implicated in the modulation of neuronal information transmission, morphological regulation, and the maintenance of synaptic function. In neurodegenerative diseases like Alzheimer’s, the aberrant expression and functional disruption of Zyxin may be linked to the initiation and progression of the disease (Lanni et al., 2013). This suggests a novel avenue for therapeutic interventions in neurological disorders.

Overall, Zyxin serves as a potential therapeutic target in various fields, including cancer, cardiovascular diseases, fibrotic disorders, inflammatory conditions, and neurological disorders. However, it also faces challenges, requiring further in-depth research and validation.

### 4.2 Strategies to address challenges in targeting zyxin as a therapeutic agent

While zyxin holds significant therapeutic potential, several challenges need to be addressed to effectively harness its benefits in clinical settings. We propose the following strategies to overcome these challenges:

#### 4.2.1 Specific targeting

Given zyxin’s multifunctionality within cells, developing specific targeting strategies is imperative. Future research should focus on elucidating the regulatory mechanisms of zyxin’s different functions, enabling the design of precise therapeutic interventions with minimal off-target effects.

#### 4.2.2 Safety and side effects

Comprehensive safety assessments are essential to mitigate potential side effects associated with zyxin-targeted therapies. Research efforts should prioritize the development of treatments that selectively modulate zyxin activity without adversely affecting normal physiological functions.

#### 4.2.3 Optimized drug delivery

Efficient drug delivery systems are crucial for the successful implementation of zyxin-targeted therapies. Continued advancements in nanotechnology and targeted delivery systems can facilitate precise drug delivery to target tissues, enhancing therapeutic efficacy while minimizing systemic side effects.

#### 4.2.4 Addressing drug resistance

Anticipating and addressing potential drug resistance mechanisms is essential for the long-term effectiveness of zyxin-targeted therapies. Research endeavors should focus on elucidating the underlying mechanisms of drug resistance and developing combination treatment strategies to mitigate its impact.

#### 4.2.5 Precision medicine approach

Embracing a precision medicine approach can optimize zyxin-targeted therapies by tailoring treatment strategies to individual patient characteristics. Integrating genomic, proteomic, and clinical data can enable personalized therapeutic interventions, maximizing treatment efficacy and minimizing adverse effects.

#### 4.2.6 Clinical validation

Rigorous clinical validation is paramount to establish the efficacy and safety of zyxin-targeted therapies in real-world clinical settings. Large-scale clinical studies are needed to validate the therapeutic benefits of zyxin modulation across diverse patient populations and disease contexts.

By implementing these strategies, we can overcome the challenges associated with zyxin-based therapeutic interventions and unlock its full potential for the treatment of various diseases**.**

## 5 Conclusion and outlook

After extensive research on zyxin, its pivotal role in cell biology and disease pathogenesis is evident. Zyxin regulates cell structure, adhesion, and signaling pathways, impacting diseases like cancer, cardiovascular issues, fibrosis, and inflammation. Zyxin holds promise in cardiovascular disease, tissue repair, cancer therapy, and neuroscience. Overcoming challenges such as specific targeting, safety, drug delivery, and resistance is crucial. In conclusion, zyxin offers broad therapeutic prospects. Addressing challenges through research and precision medicine applications aims to realize its full potential, enhancing treatment options. Continued scientific advancements will further explore zyxin’s therapeutic avenues, offering hope for medical progress.
